# Canadian Association of Gastroenterology Communique: After-Hours Endoscopy Cart

**DOI:** 10.1093/jcag/gwz032

**Published:** 2019-11-21

**Authors:** Mandip Rai, Mary Cooper, Scott Shulman, Dan Kottachchi, Sandra Nelles, Mark Macmillan, Steven Heitman, Alan Barkun, Frances Tse, Lawrence Hookey

**Affiliations:** 1 Division of Gastroenterology, Queen’s University, Kingston, Ontario, Canada; 2 Division of Gastroenterology, North Ontario School of Medicine, Thunder Bay, Ontario, Canada; 3 Division of Gastroenterology, North Bay Regional Health Centre, North Bay, Ontario, Canada; 4 Division of Gastroenterology, Guelph General Hospital, Guelph, Ontario, Canada; 5 Division of Gastroenterology, Trillium Health Partners, Mississauga, Ontario, Canada; 6 Division of Gastroenterology, Dalhousie University, Memorial University, Fredericton, New Brunswick, Canada; 7 Division of Gastroenterology, University of Calgary, Calgary, Alberta, Canada; 8 Division of Gastroenterology, McGill University and the McGill University Health Centre, Montreal, Quebec, Canada; 9 Division of Gastroenterology, McMaster University, Hamilton, Ontario, Canada; 10 Gastrointestinal Diseases Research Unit, Department of Medicine, Queen’s University, Kingston, Ontario, Canada

**Keywords:** After-hours endoscopy, Therapeutic endoscopy, Upper gastrointestinal bleed

## Abstract

**Background:**

Endoscopic procedures performed after-hours often require therapeutic interventions that are technically demanding for the endoscopist. The aim of this position paper is to provide guidance on the minimum standard of equipment that should be available on a mobile endoscopy cart for provision of a safe and effective after-hours emergency endoscopy service. The guidance is based on consensus among academic and community gastroenterologists in Canada.

**Methods:**

A modified Delphi process was used to establish consensus among 9 participants. A list of statements was prepared by an expert panel of endoscopists. The statements were divided into three broad sections for what should be on an after-hours endoscopy cart including medications, nonendoscopic tools and therapeutic/diagnostic equipment. Consensus for being on the endoscopy cart was achieved when 75% or more of voting members indicated ‘agree’.

**Results:**

For nonendoscopic tools, there was agreement for having sterile saline, sterile water, endoscope lubricant, various syringes, bite blocks (paediatric and adult size), a water pump with foot peddle, formalin jars for biopsy specimens, digital photo and printing capability and an overtube. For medications, there was agreement for having hyoscine butylbromide and epinephrine on the cart. For therapeutic/diagnostic tools, there was agreement for having biopsy forceps (standard and jumbo), polypectomy snares, sclerotherapy needles and agent (for a variceal bleed), band ligation kit, multipolar electrocautery probes, heater probe catheter, endoscopic clips, hemostatic powder and retrieval devices.

**Interpretation:**

This position paper provides guidance on the minimum standard of items that should be on an after-hours endoscopy cart. Standardization of equipment may help improve safety and quality of after-hours endoscopic procedures.

## Introduction

It is not uncommon to need to perform therapeutic endoscopy after-hours. This requires a significant clinical and economic resource investment for the system. Acute upper gastrointestinal bleeds, foreign body ingestion and food bolus impaction are commonly encountered after hours. These cases can be technically challenging and require expertise and support. A survey of the practice of after-hours and emergency endoscopy in Canada revealed that 25% of adult gastroenterologists had no on-call endoscopy nurse (1). Despite clear recommendations on the management of conditions requiring urgent endoscopy (2–4), there lacks guidance on what equipment is needed on an after-hours endoscopy cart.

There is literature to guide the design of endoscopy units adequately equipped to perform high quality diagnostic and therapeutic endoscopy services (5,6). After-hours endoscopic procedures, however, are commonly completed outside of the endoscopy unit including in the emergency room, intensive care unit or the operating suite. To complete these procedures safely, effectively and efficiently, substantial planning and preparation is required. Most centres have a mobile endoscopy cart for these situations that includes equipment and devices necessary to perform therapeutic intervention. However, no standard exists to inform the make up of an after-hours endoscopy cart. Thus, the aim of this study was to understand what is needed on an after-hours endoscopy cart with the goal of establishing a checklist for equipping these carts to support safe and effective on call endoscopic care.

## Methods

**Figure 1.**
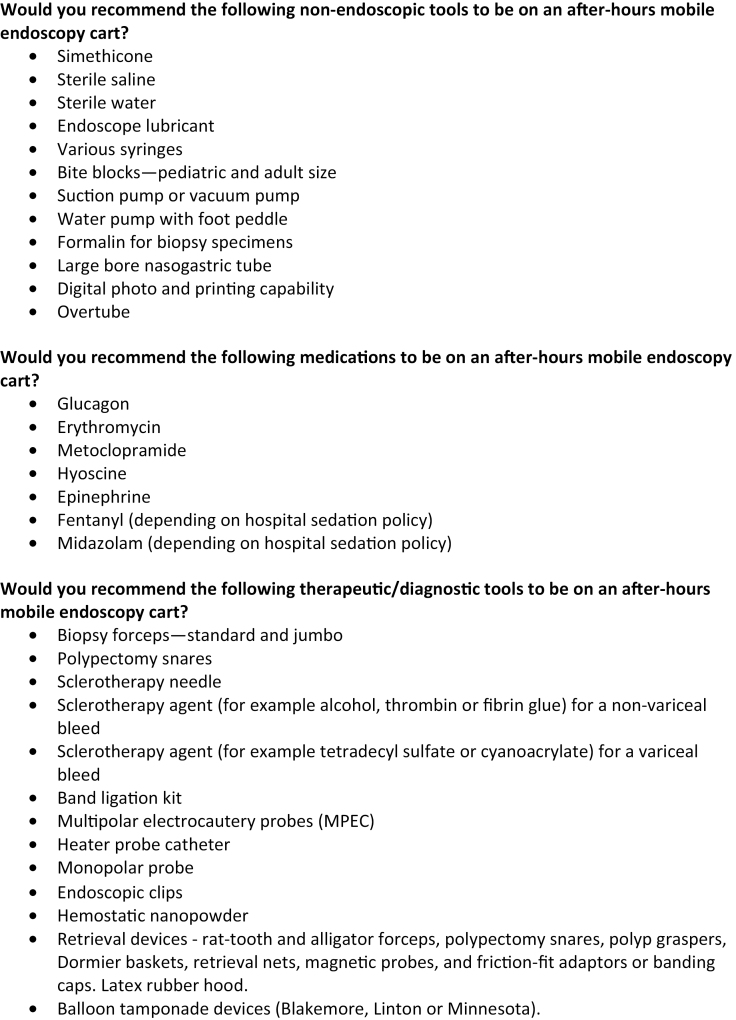
The questionnaire provided to participants which was comprised of 31 recommendations. Participants were asked if they agreed or disagreed with each statement.

A modified Delphi process was performed to establish the consensus for what should be on an after-hours endoscopy cart. This process was based on an anonymous single round voting system. A list of statements was initially prepared by an expert panel of endoscopists. This panel consisted of three Canadian academic therapeutic staff gastroenterologists with over 10 years of experience as staff. A total of nine endoscopists with at least 5 years of experience as a staff gastroenterologist in Canada who perform therapeutic endoscopy after hours were asked to complete the survey. This group of nine endoscopists included the expert panel. The statements were divided into three broad sections including medications, nontherapeutic and therapeutic/diagnostic equipment (Figure 1).

The questionnaire was comprised of 31 questions. Participants were asked if they agreed or disagreed with each question. The data were summarized by calculating mean scores for each of the questions. Consensus for being on the endoscopy cart was achieved when 75% or above of voting members indicated ‘agree’. Consensus for not being on an endoscopy cart was achieved when 75% or above of voting members indicated ‘disagree’. Participants were also asked to provide comments for each question and to suggest additional supplies they felt were pivotal for an after-hours endoscopy cart, how their endoscopy carts are stocked and challenges they face with after-hours endoscopy.

The expert panel reviewed the results and co-authors were involved in the final editing of the commentaries, consensus statements and the final manuscript.

## Results

**Figure 2.**
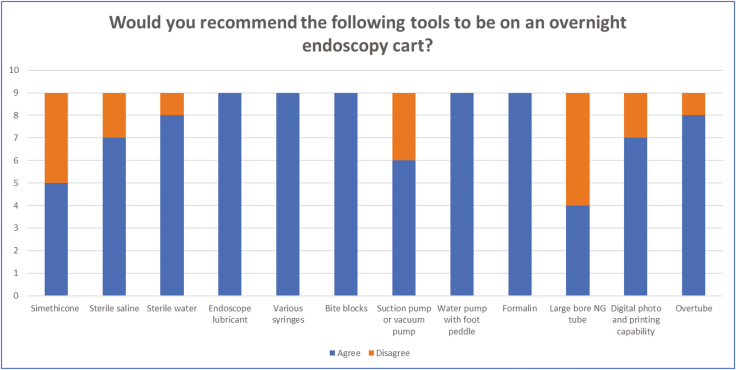
Results from the first question which looked at what nonendoscopic tools should be on an after-hours mobile endoscopy cart. There was at least 75% agreement for having sterile saline, sterile water, endoscope lubricant, various syringes, bite blocks (paediatric and adult size), water pump with foot peddle, formalin for biopsy specimens, digital photo and printing capability and an overtube.

**Figure 3.**
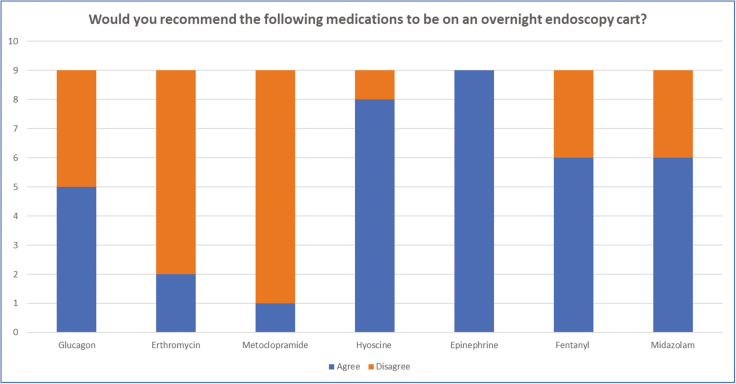
Results from the second question which looked at what medications should be on an after-hours mobile endoscopy cart. There was at least 75% agreement on having hyoscine and epinephrine on the cart. There was at least 75% of people disagreeing with having erythromycin and metoclopramide.

**Figure 4.**
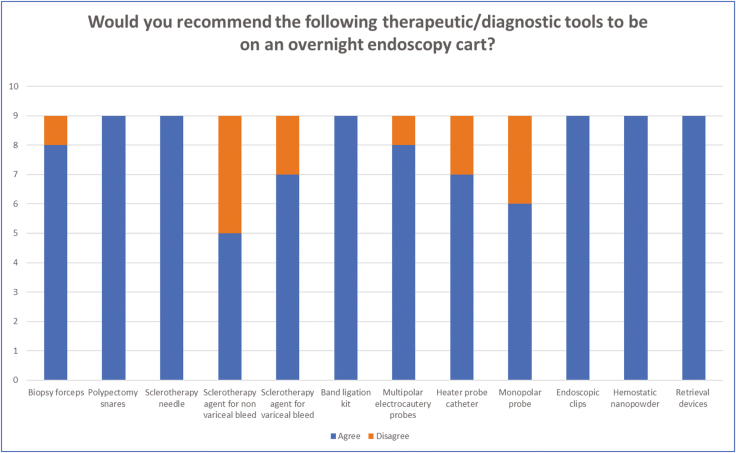
Results from the third question which looked at what therapeutic/diagnostic tools should on an after-hours mobile endoscopy cart. There was at least 75% agreement for having biopsy forceps (standard and jumbo), polypectomy snares, sclerotherapy needle and agent for a variceal bleed, band ligation kit, multipolar electrocautery probes, heater probe catheter, endoscopic clips, hemostatic nanopowder and retrieval devices.

Nine endoscopists from different institutions completed the survey (Figures 2–4). Four practice at tertiary academic centres and five practice at a community hospital. Six practices in Ontario, one in Quebec, one in Alberta and one in New Brunswick.

### Section One: Nonendoscopic Tools on an After-hours Endoscopy Cart

There was at least 75% agreement for having sterile saline, sterile water, endoscope lubricant, various syringes, bite blocks (paediatric and adult size), a water pump with foot peddle, formalin jars for biopsy specimens, digital photo and printing capability and an overtube. Consensus was not achieved on having simethicone, a suction or vacuum pump and a large bore nasogastric tube.

### Section Two: Medications on an After-hours Endoscopy Cart

There was at least 75% agreement for having hyoscine butylbromide and epinephrine on the cart. At least 75% disagreed with having erythromycin and metoclopramide. Consensus was not achieved on glucagon, fentanyl or midazolam.

### Section Three: Therapeutic/Diagnostic Tools on an After-hours Endoscopy Cart

There was at least 75% agreement for having biopsy forceps (standard and jumbo), polypectomy snares, sclerotherapy needles and agent for a variceal bleed, band ligation kit, multipolar electrocautery probes, heater probe catheter, endoscopic clips, hemostatic powder and retrieval devices. Consensus was not achieved on having a monopolar probe, sclerotherapy agent for a nonvariceal bleed and balloon tamponade devices.

Additional details on the results of the survey are provided in the Supplementary Appendix.

## Interpretation

Through a structured consensus process, this study looked at what should be included on an after-hours endoscopy cart. The expert panel was comprised of experienced gastroenterologists who perform therapeutic endoscopy after-hours. Thus, this position paper provides some clarity on an important clinical topic based on expert opinion. To our knowledge, no prior studies have assessed what is needed on an after-hours endoscopy cart.

The first question addressed standard equipment. Not unexpectedly, consensus was achieved for the basic supplies needed to perform endoscopy including sterile saline, sterile water, endoscope lubricant various syringes, bite blocks, a water pump with foot peddle, formalin jars for biopsy specimens, digital photo and printing capability and an overtube. Overtubes are essential as they may be needed to protect the airway during removal of foreign objects. Furthermore, they allow easy passage of the endoscope for removing multiple objects while protecting the esophageal mucosa from sharp objects.

Consensus was not achieved on having simethicone, a suction or vacuum pump and a large bore nasogastric tube. The utility of a nasogastric tube for suspected gastrointestinal bleeding is debatable and has fallen out of clinical practice in many centres. Its use has not been shown to reduce mortality, length of hospital stay, surgery or transfusion requirement (7). If needed, they are usually readily available throughout the hospital. Simethicone is commonly used during endoscopy to improve mucosal visibility. The safety of the use of simethicone, however, has been called into question based on a report by Ofstead et al. that demonstrated the presence of simethicone in endoscopes following reprocessing and, thus, the theoretical potential for biofilm formation (8). The Canadian Association of Gastroenterology recommends reconsidering the routine use of simethicone during endoscopic procedures and, when needed, to use the lowest effective dose (9). It is not surprising that suction or vaccum pumps were not considered necessary given after-hours endoscopy is usually performed in the intensive care unit, operating suite or emergency department where these devices are usually readily available at the bedside.

The second question evaluated medications. Consensus was achieved for hyoscine butylbromide and epinephrine on an overnight cart. Hyoscine works by causing smooth muscle relaxation to reduce spasms in the gastrointestinal tract. Cardiac monitoring is essential when administering this drug as it can be associated with tachycardia and hypotension. Epinephrine injection therapy favours hemostasis by creating local tamponade and possibly by causing local vasospam. Consensus was not achieved on having erythromycin and metoclopramide on the travel cart. Prokinetics are used to improve mucosal visualization at the time of gastroscopy. A meta-analysis of erythromycin versus placebo in patients with upper gastrointestinal bleeding demonstrated the incidence of an empty stomach at the time of endoscopy was significantly increased in those who received erythromycin (10). In addition, the need for a second endoscopy, amount of blood transfusions and the length of hospital stay were significantly reduced. In order to be effective, these medications must be given in advance of endoscopy (30 to 90 minutes prior) and thus, the utility of having them on an endoscopy cart is low.

Consensus was not achieved as to the need for storing glucagon, fentanyl or midazolam on the endoscopy cart. Sedation medications are usually kept in a secure area as per hospital policy and thus having them on an endoscopy travel cart is ill-advised and may be against institutional policies. As most overnight endoscopy is performed in the emergency department, operating room or intensive care unit, these medications are readily available. Glucagon can be used to attempt to relax the esophagus in the setting of a food bolus. Studies have shown mixed results and if anything glucagon would have been attempted prior to requesting an after-hours procedure (11,12).

In the third question, consensus was achieved for having biopsy forceps (standard and jumbo), polypectomy snares, sclerotherapy needle, band ligation kit, multipolar electrocautery probes, heater probe catheter, endoscopic clips, a hemostatic powder kit and retrieval devices. Tc-325 is the only approved hemostatic powder in Canada and is an inorganic biologically inert compound that has granules which, when in contact with moisture in the gastrointestinal tract, become coherent and adhesive to an actively bleeding surface, serving as a mechanical barrier for hemostasis (13). Tc-325 may also act through playing a role in clot formation and shortening coagulation time (14). In a multicentre prospective study looking at the use of Tc-325 in nonvariceal upper gastrointestinal bleeding as a monotherapy, 85% achieved primary hemostasis with a rebleeding rate of 15% at 7 days (15). It is probably best used as a rescue therapy in general, but should not be used alone in peptic ulcer bleeding. Its possible benefits have also been shown in malignant gastrointestinal bleeding (16). Having a sclerotherapy needle on the cart is essential as it can be used for epinephrine or saline for nonvariceal lesions and sclerosant for varices.

Consensus was not achieved on having a monopolar probe and balloon tamponade devices. As per the Baveno VI Consensus, balloon tamponade should only be used in refractory esophageal variceal bleeding as a temporary bridge until definitive treatment can be instituted (17). Endoscopists performing overnight endoscopy should have access to a balloon tamponade device in case of an emergency. If not stored on the travel cart, it should be kept in an area that can quickly be accessed. If there is suspicion of a variceal bleed, then it should be readily available along with endoscopic glue in case of gastric varices.

Consensus was achieved for having a sclerotherapy agent for a variceal bleed (for example tetradecyl sulfate or cyanoacrylate). Consensus was not achieved for having a sclerotherapy agent for a nonvariceal bleed (e.g., alcohol, thrombin or fibrin glue). Data exist to support the use of sclerosant injection in both variceal and nonvariceal bleeds (18). However, it is rarely used for nonvariceal bleeding. Endoscopic variceal ligation therapy has replaced sclerotherapy as the preferred treatment for bleeding esophageal varices but sclerotherapy is still used under certain circumstances for esophagogastric varices. Certain agents need to be kept refrigerated and so being accessible after hours is important. Another factor is if the endoscopist is trained in using the sclerotherapy agents. If it is a facility where most endoscopists are trained then it might be more reasonable to be on the cart all the time.

We acknowledge the limitations of this position paper. The recommendations were developed through a consensus method and thus represents the opinion of the participants. Generalizability may be restricted as there were a limited number of participants and a limited number of centres from across Canada. Despite these issues, our paper serves to increase awareness and promote conversations among stakeholders to ensure appropriate resources are available. Not only should the endoscopy cart be stocked appropriately, all health care personnel involved in performing the procedure should know how to use and trouble shoot problems with the equipment.

In summary, after-hours endoscopy is commonly performed. Through a structured consensus process, we provide guidance for what should be on an after-hours endoscopy cart. Standardization of equipment may help improve safety and quality of after-hours endoscopic procedures.

## Notes

*Funding:* No funding was received to complete this study.
